# Progress and Prospects in the Treatment of Lacrimal Gland Dysfunction Diseases: From Traditional Treatment Methods to Stem Cell and Organoid Therapies

**DOI:** 10.1155/sci/6334284

**Published:** 2025-08-08

**Authors:** Xiaona Chen, Shixu Li, Yongxin Zhang, Lin Ye

**Affiliations:** ^1^Shenzhen Eye Hospital, Shenzhen Eye Hospital Affliated to Jinan University, Shenzhen 518040, China; ^2^Shenzhen Eye Hospital, Shenzhen Eye Medical Center, Southern Medical University, Shenzhen 518040, China; ^3^Department of Ophthalmology, The Second Hospital of Shandong University, Jinan, China

**Keywords:** lacrimal gland (LG) dysfunction, organoid therapy, stem cell therapy, three-dimensional (3D) bioprinting technology, traditional treatment methods

## Abstract

Lacrimal gland (LG) dysfunction diseases are a type of disorder caused by various etiologies that damage the LG tissue, reducing lacrimal fluid secretion, triggering aqueous-deficient dry eye (ADDE), and causing a series of complications like keratoconjunctivitis sicca, potentially threatening vision. Our review summarizes the limitations and new progress of traditional treatment methods for LG dysfunction diseases. Meanwhile, we conduct in-depth analyses closely centered on the two emerging and cutting-edge research hotspots, namely stem cell therapy and organoid therapy. We have comprehensively evaluated the current research status regarding various stem cells, their derived extracellular vesicles, and LG organoid transplantation, further discussed the existing deficiencies, and subsequently put forward the prospective directions for future research. These include developing ophthalmic preparations of extracellular vesicles and LG stem cells or searching more efficient drug delivery systems, as well as culturing LG organoids that are highly similar to human lacrimal glands (LGs) in both function and microstructure through magnetic three-dimensional (3D) bioprinting technology and microfluidic 3D bioprinting technology.

## 1. Lacrimal Gland (LG) Dysfunction Diseases

LG dysfunction diseases are a type of disorder caused by various etiologies that damage the LG tissue, reducing lacrimal fluid secretion, triggering aqueous-deficient dry eye (ADDE) and causing a series of complications like keratoconjunctivitis sicca, potentially threatening vision. According to reports, the prevalence rate of dry eye disease worldwide ranges from 5% to 50%, with ADDE caused by LG dysfunction accounting for approximately one-third of all dry eye diseases [1]. What's more, since ADDE can cause severe ocular surface inflammation and chronic corneal diseases, its condition is often more serious and has a potential risk of leading to blindness [2]. The LG, whose main function is to generate tears, serves as the main source of liquids, electrolytes, and proteins within tears [3]. Tears serve not only to maintain the moisture of the ocular surface but also, fulfill a variety of crucial functions including lubrication, cleansing, nourishment, and antibacterial properties. They can effectively protect the ocular surface from external stimuli and infections and ensure the integrity and normal metabolism of the corneal epithelium. When the function of the LG is disrupted, a significant reduction in tear secretion volume or an abnormal alteration in tear composition occurs. Consequently, the lubricating and protective mechanisms of the ocular surface are compromised, thereby precipitating dry eye disease. Research on treatments for LG dysfunction diseases is anticipated to restore normal LG secretion, rebuild a healthy ocular surface microenvironment, decrease the incidence of dry eye disease and its complications, and enhance patients' quality of life in the future.

The LG can be divided into the main LG and the accessory lacrimal glands (LGs). The main LG is situated in the lacrimal fossa of the superotemporal orbit and is divided into the orbital lobe and the palpebral lobe by the aponeurosis of levator palpebrae superioris muscle. The accessory LGs are distributed near the conjunctival fornix and mainly include Krause's glands and Wolfring's glands. The acinus, being the basic secretory unit of the LG, is surrounded by single-layer cubic or columnar epithelial cells with secretory functions. It can extend ducts, which gradually converge to form branching ducts at various levels and finally terminate into wide excretory ducts that open onto the ocular surface. The orbital lobe and palpebral lobe of the main LG each have their own excretory ducts that open respectively on the lateral part of the superior fornix conjunctiva and are the major structures influencing the secretion of tears. Meanwhile, the accessory LGs also have their own independent small ducts which open on the conjunctival surface, as well, and they cooperate with the main LG to complete the drainage of tears. When stimulated by factors, such as nerves or hormones, the acinar cells secrete the processed components of tears into the acinar cavity through exocytosis, and then these components are discharged onto the ocular surface through the duct system [4, 5]. Therefore, when structures, such as the acini and ducts of the LG are damaged or when the stimulating factors like nerves and hormones are abnormal, the normal process of tear secretion will be disrupted, thereby leading to ADDE.

Depending on the underlying etiology, LG dysfunction diseases are classified into inflammatory and noninflammatory types. The inflammatory type is more common, including Sjögren's syndrome, Stevens-Johnson syndrome, mucous membrane pemphigoid, graft-versus-host disease, ocular chemical burns, corneal refractive surgeries, long-term use of topical and systemic medications, and so on. The most common mechanism is that autoimmunity targets the LG, causing activated T cells to infiltrate the gland, which then leads to diffuse glandular atrophy with the death of acinar and duct cells, as seen in Sjögren's syndrome [6]. In addition, the long-term presence of chronic inflammation or injury in the conjunctiva can cause cicatricial proliferative conjunctivitis, and the continuous subconjunctival fibrosis will also lead to the destruction of the lacrimal canaliculi, but generally, it does not affect the acini [7, 8]. The noninflammatory type is less prevalent and includes primary, congenital, paralytic, and toxic cases, along with the radiotherapy of head and neck cancers and hormonal levels [9, 10].

Patients with LG dysfunction diseases will typically initially experience symptoms, such as ocular dryness, a sense of foreign body presence, and a burning discomfort. As the progression of the disease unfolds, complications like keratoconjunctivitis sicca will exacerbate, and the cornea may develop opacities and ulcers, thereby further endangering ocular health and vision. In clinical practice, the dynamic assessment of tear secretion (DATS) [11] and the evaluation of tear meniscus height (TMH) [12] are commonly used for the diagnosis of LG dysfunction diseases. Moreover, in vivo confocal microscopy can reveal the density of acinar units, the diameter of acinar units, and the density of inflammatory cells in the damaged LG, directly reflecting the changes in the structure and cellular state of the LG after being damaged, which is helpful for etiological judgment [13, 14].

**Figure 1:**
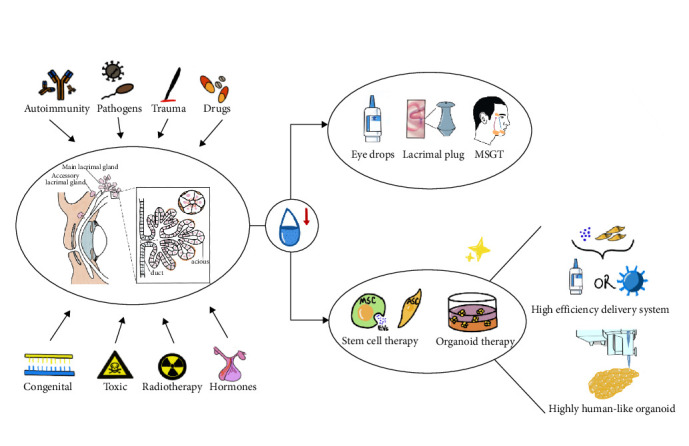
Progress and prospects in the treatment of LG dysfunction diseases.

Although, LG dysfunction diseases are among the most common ocular surface diseases, there is currently no definitive treatment available. Most of the traditional treatment methods are palliative, including artificial tears, secretagogues, and anti-inflammatory drugs. Despite the emergence, of some new advances, specific treatment approaches are still lacking. In recent years, stem cell and organoid therapies have achieved certain progress in the field of LG regeneration, which are expected to break through the limitations of traditional treatment methods and restore the structure and function of the LG fundamentally. Thus, the purpose of this review is to clarify the limitations and new advances of traditional treatment methods for LG dysfunction diseases and summarize the possible future treatment strategies. It mainly focuses on the two emerging and frontier research hotspots, stem cell and organoid therapies, hoping to provide directions for further research and discussion (Figure 1).

## 2. Traditional Treatment Methods for LG Dysfunction Diseases

Currently, various treatment methods have been developed clinically for LG dysfunction diseases to relieve the symptoms of insufficient tear secretion or improve the secretory function of the LG, and with the continuous progress of medical research in recent years, many new advances have emerged.

### 2.1. Traditional Treatment Methods for Alleviating the Symptoms of Insufficient Tear Secretion

#### 2.1.1. Artificial Tears

Artificial tears, of which the principal constituent is hyaluronic acid, are characterized by their ease of use, accessibility, and relative safety. Commonly, they are included in the first-line treatment protocols for all kinds of dry eyes [15]. A sterile packaging design with a one-way outlet has been developed to reduce the toxic effects of preservatives during long-term use [16]. Evidence indicates that applying artificial tears around four times daily can alleviate the symptoms of dry eye disease within 1 month of regular use. If there is no benefit after 1 month, other alternative treatment methods should be taken into consideration [17]. As a palliative treatment, artificial tears are only applicable to patients with mild LG dysfunction, serving to temporarily relieve discomfort symptoms. They have no effect on improving the secretory function of the LG and the duration of effect is limited.

#### 2.1.2. Lacrimal Plugs

Lacrimal plugs can prolong the time when tears remain on the surface of the eyes, which helps to relieve symptoms and reach good curative results [18]. Lacrimal plugs can be divided into punctal plugs and canalicular plugs according to their location, and they can be classified as, temporary and permanent according to the time of their placement [19]. Permanent plugs, which stay longer, usually have a greater impact than degradable ones and are more likely to cause side effects, such as lacrimal canaliculitis and pyogenic granuloma. Punctal plugs are well tolerated with only about 10% needing removal due to irritation, yet the probabilities of epiphora and plug loss are higher than those of canalicular plugs [20]. Smart plug punctal plugs have been used clinically for many years [21]. The new OASIS preloaded punctal plugs, by contrast, have a similar efficacy and can cut down on the loss of plugs before implantation and the abnormal implantation caused by an increase in plug amount [22]. In sum, while punctal plugs are applicable for treating ADDE with some efficacy, they fail to fundamentally enhance LG function. Moreover, risks like ocular discomfort, infection, plug displacement or loss exist, thus limiting their clinical application.

### 2.2. Traditional Treatment Methods for Improving the Secretory Function of the LG

#### 2.2.1. Biological Tear Substitutes

Compare with artificial tears, biological tear substitutes are remarkably similar to the biochemical properties of natural tears produced by the LG, such as osmolarity and pH value. In addition, they also contain growth factors, vitamins, enzymes and other substances, which provide the nutrients necessary for epithelial healing [23]. Serum-based products include autologous serum and cord blood serum, and platelet-derived products include platelet-rich plasma, plasma rich in growth factors, and platelet lysate. Meta-analyses have shown that biological tear substitutes are superior to artificial tears and secretagogues [24]. A recent study has also proven that, adding growth factor-rich plasma to the standard treatment of artificial tears combined with anti-inflammatory agents can improve the ocular symptoms and signs of inflammation in patients with moderate to severe conditions [25]. However, the preparation and efficacy of biological tear substitutes differ significantly. These differences stem from varying sources (autologous and allogeneic), processing techniques (centrifugation, activation, and lysis), and the resulting growth factor profiles, which in turn lead to divergent clinical outcomes, such as disparities in repair kinetics and immunogenic risks. There is still a lack of evaluation of long-term curative effects and standardized preparation protocols for various biological tear substitutes [26]. Furthermore, the high preparation cost and the lack of storage facilities have limited its use.

#### 2.2.2. Anti-Inflammatory Drugs

Commonly used anti-inflammatory drugs in clinical practice, such as topical corticosteroids, nonsteroidal anti-inflammatory drugs, have side effects and are not suitable for long-term use. While cyclosporine has no significant side effects, its long-term use incurs high costs. Moreover, due to the significant differences in patient responses, the treatment is complex and uncertain. Often, it is necessary to combine other methods to improve the curative effect and reduce the recurrence rate [27–29]. In recent years, more efficient, safe, and targeted ocular drug delivery systems based on nanotechnology are expected to overcome the limitations of anti-inflammatory drugs in treating eye diseases [30]. Lifitegrast nanoparticles were approved by the FDA in 2016. Additionally, a preservative-free form of cyclosporine nanoparticles has also been approved recently [31, 32]. The effectiveness of subconjunctival dendrimer-dexamethasone therapy in the treatment of dry eye disease has been studied and is currently in the preclinical stage [33]. Recently, a cationic polypeptide micelle MTem/Los conjugated with a specific inhibitor and a scavenger has also been reported. It can jointly inhibit the inflammation pathways through multiple targets, possess good biocompatibility, effectively encapsulate drugs, enhance the curative effect and reduce the dosing frequency, thus providing new ideas for designing more efficient and safe therapeutic agents for ocular surface inflammatory diseases [34]. However, due to practical application problems, such as high research and development costs, complex preparation processes, and great difficulties in quality control, the ocular drug delivery systems based on nanotechnology have not been widely used in clinical practice yet.

#### 2.2.3. Secretagogues

The most commonly used secretagogue, pilocarpine, which stimulates the M3 receptors of exocrine glands, has its tablet and eye drop formulations for patients with dry eye disease [35, 36]. Cevimeline is similar to pilocarpine, but it has better cardiopulmonary safety than pilocarpine and is more frequently used abroad [37]. In addition, there are also some rare or under-research secretagogues. Diquafosol tetrasodium 3% ophthalmic solution acts on the P2Y2 receptors on the corneal epithelium, conjunctival epithelium, goblet cells and meibomian glands, increases the intracellular calcium, and promotes the secretion of the aqueous and mucin components in the tear fluid, which has been approved for use in Japan and South Korea [38]. Rebamipide acts on goblet cells to increase mucin production and modulates PGE2, PGI2, and TNF-A levels to prevent macrophage infiltration, reduce ocular surface inflammation and further enhance tear film stability [39]. It has been reported to be used for dry eye disease related to chronic graft-versus-host disease [40]. The significant efficacy of Lacripep (a synthetic lacrimal protein fragment) eye drops in increasing tear secretion, proven by clinical studies, is undergoing further research regarding appropriate doses and concentrations [41]. Recombinant human nerve growth factor eye drops can promote corneal healing, enhance conjunctival epithelial differentiation and mucin secretion, which is undergoing Phase III clinical trials [42, 43]. Nevertheless, the long-term application of secretagogues might exert unfavorable impacts on the secretory tissues of the LGs, resulting in a reduction or even exhaustion of the secretory function. Additionally, it could trigger other adverse reactions either in the eyes or throughout the body. Consequently, safety research regarding long-term usage remains to be carried out.

#### 2.2.4. Salivary Gland Transplantation

Salivary gland transplantation, using the salivary gland as functional exocrine tissue, aims to reconstruct a stable tear film and is suitable for patients with severe LG dysfunction. Some studies have suggested, that sealing the graft while retaining the overlying mucosa and submucosal tissue could solve the problem of postoperative subconjunctival fibrosis [44]. The salivary glands used as substitutes for the LGs include three major salivary glands and the minor salivary glands [45]. At present, there is a stronger preference for performing transplantation using the submandibular gland and the labial gland. Among them, the labial gland has the best long-term outcomes [46]. The crucial steps before the surgery are the measurement of the flow rate of minor salivary glands and the selection of the donor site. Staining the labial mucosa with fluorescein strips can objectively assess the number of functional openings and the secretion rate of the ocular surface graft [47]. Patients with salivary gland hypofunction can undergo allogeneic transplantation, and with the assistance of bone marrow mesenchymal stem cell (MSC) treatment and FK506, a higher survival rate is expected to be achieved [48, 49]. However, currently, the effectiveness of salivary gland transplantation is limited and the recovery of visual acuity is controversial. Only 46% of patients with labial gland transplantation showed improved vision. This may be due to the abnormal composition of tears after transplantation. Compared to normal tears, the “salivary tears” have a low concentration and low total protein content, and their electrolyte and biochemical compositions are also largely unclear [50].

In conclusion, owing to their respective limitations, traditional treatment methods have failed to achieve favorable therapeutic effects. Therefore, there is an urgent need for a treatment method that can restore the structure and function of the LG fundamentally. Over the recent years, many scholars have attempted to apply stem cell and organoid therapies and have made certain progress.

## 3. Stem Cell Therapy

The applications of in vitro expanded stem cells in regenerative medicine rely not only on their differentiation potential, but more importantly on their immunomodulatory ability to create a favorable immune microenvironment and release growth factors to activate endogenous tissue repair [51].

### 3.1. Direct Transplantation of Stem Cells and Stem Cell Products

#### 3.1.1. Mesenchymal Stem Cells (MSCs)

Bone marrow MSCs were the first to be discovered and extracted, and they have been widely studied over the years. Lee et al. [52] applied bone marrow MSCs by injecting them periorbitally into model mice with concanavalin A-induced inflammation-mediated LG dysfunction. They found that this could reduce the infiltration of CD4+ T cells and the levels of inflammatory cytokines in the intraorbital glands and on the ocular surface. Moreover, it significantly increased the production of aqueous tears and the number of conjunctival goblet cells. Furthermore, Aluri et al. [53] found that bone marrow MSCs could not only relieve inflammation but also increase the expression of aquaporin 5. However, bone marrow-derived MSCs are not easily obtained. Donors may suffer damage and pain while the extraction and preparation processes are hard to have quality control over, and immune reactions may be induced when they are transplanted into allogeneic recipients [54]. These drawbacks have limited the clinical applications of bone marrow MSCs.

Adipose-derived MSCs have the advantage of being the easiest and most abundant MSCs type to acquire from adult tissue [54]. Li et al. [55] implanted adipose-derived MSCs into rabbit models of ADEE and found that these cells improved autoimmune dacryoadenitis by regulating the Th1/Th17 response, thus promoting the repair of the LGs. Subsequently, Villatoro et al. [56] transplanted allogeneic adipose-derived MSCs around the LGs in dogs with ADEE. For the first time, it was proved that the allogeneic adipose-derived MSCs were well tolerated, had no adverse reactions, and effectively improved the secretory function of the LGs. Furthermore, Bittencourt et al. [57] demonstrated that a single dose of a low number of allogeneic adipose-derived MSCs can be utilized for the treatment of severe keratoconjunctivitis sicca in dogs, and exerts its effect over an extended period, which can last up to 12 months, even with just a single administration. Recently, Møller-Hansen et al. [58] conducted a double-blind, randomized clinical trial involving 54 patients with dry eye disease secondary to Sjogren's syndrome. It was proved that injecting allogeneic adipose-derived MSCs into the LGs of patients with ADEE is a safe and feasible treatment method for severe ADDE.

Umbilical cord MSCs exist in the umbilical cord tissue of newborns. They have a higher cell content, stronger proliferative ability and lower immunogenicity. Moreover, they possess advantages, such as convenient sourcing of materials and the absence of ethical controversies. Therefore, they are attracting increasing attention from researchers [59]. Lu et al. [60] investigated the therapeutic effects of human umbilical cord MSCs on rabbit models of autoimmune dacryoadenitis. They found that these cells could alleviate the ongoing autoimmune dacryoadenitis by polarizing macrophages into an anti-inflammatory phenotype through influencing the AKT pathway. However, at present, the research on the transplantation of human umbilical cord MSCs in terms of LG repair is still insufficient, and it is urgently necessary to conduct further in-depth explorations.

LG specific MSCs seem to be superior to ectopic MSCs for the treatment of ADDE, since the tissue-specific differences between the subpopulations have been described. Dietrich et al. [61] transplanted LG specific MSCs into the damaged LGs of mouse models of ADDE induced by oviduct ligation. It was observed that the number of acinar structures was significantly increased compared to that of the control group, and the immune reaction was also modulated. This proves that the application of LG specific MSCs seems to be a promising therapeutic approach. However, the problems of its limited source and the great difficulty in isolation and culture urgently need to be solved.

However, as a type of pluripotent stem cells derived from the mesenchyme of the embryonic mesoderm, MSCs possess the abilities of self-renewal and multidirectional differentiation, but they also have uncertainties. Yan et al. [62] proposed that MSCs are a double-edged sword in tumorigenesis. They may either inhibit tumorigenesis or promote tumor metastasis and tumorigenesis, and their immunomodulatory role in the tumor microenvironment is complex and unknown. Meanwhile, Eiro et al. [63] believed that the outcome of treatment may depend on the source site of MSCs and the type of tumor involved. These factors have significantly limited their clinical applications.

#### 3.1.2. Extracellular Vesicles Derived From MSCs

Extracellular vesicles are produced by MSCs. Compared with MSCs, extracellular vesicles have lower immunogenicity, lower tumorigenicity, as well as more convenient storage and transportation, and thus have a broad application prospect [64].

Studies [64] have shown that adipose MSCs secrete high levels of extracellular vesicles, which have immunomodulatory effects. Abuganam et al. [65] used adipose MSCs and the extracellular vesicles secreted by them to treat mouse models of Sjogren's syndrome in groups. It was confirmed that the extracellular vesicles have a similar effect on promoting the proliferation and regeneration of the LG as adipose MSCs do. Similarly, Wang et al. [66] discovered that the extracellular vesicles of mouse adipose MSCs significantly increased the tear volume and the ratio of LG to body weight by activating the NGF/TrkA pathway involving dendritic cells, and also, promoted the regeneration of corneal nerves and the restoration of sensation. Subsequently, Wang et al. [67] confirmed that the extracellular vesicles derived from human umbilical cord MSCs also, have the effect of promoting the proliferation and regeneration of the LG.

However, extracellular vesicles have limitations in the field of LG regeneration, such as difficulties in separation and purification, high heterogeneity, and poor in vivo stability and bioavailability. It is urgent to solve these problems to promote their effective application in this field.

#### 3.1.3. LG Stem Cells

The ideal source of stem cells for regenerating the LG is the patient's own LG tissue, which limits the risks of immune response, transplant rejection, and tumorigenesis caused by multidirectional differentiation, and does not involve ethical issues [54].

Gromova et al. [68] isolated LG epithelial progenitor cells from the LGs of adult wild-type mice and implanted them into the LGs of mice with chronic inflammation of the LG induced by TSP-1 gene knockout. This provided the first evidence for the effective use of adult LG epithelial progenitor cell transplantation to rescue human LG dysfunction.

Mishima et al. [69] transplanted side population cells (endothelial-like cells with stem cell characteristics and being CD31 positive) isolated from the LGs of mice into model mice with irradiation-induced LG hypofunction. It was observed that the side population cells could also mediate the recovery of the hypofunctional LGs.

Ali et al. [70] reported for the first time that human accessory LG tissues contain precursor marker-positive cells and express histone-1, which indicates that they are essentially stem cells. They proposed that accessory LG stem cells might be a reliable source of stem cells for regenerating the LG, but further in-depth exploration is needed.

However, the obtained LG stem cells are limited and the cell expansion conditions are complex. For transplantation, a large number of cells need to proliferate and be continuously passaged while ensuring their viability. This has restricted the further development of LG stem cell transplantation. Chapman et al. [71] proposed the conditional reprograming technique, which involves combining irradiated Swiss 3T3-J2 mouse fibroblast feeder cells with the ROCK inhibitor Y-27632. This enables primary human keratinocytes to continuously proliferate and be effectively immortalized in vitro, thus nicely solving this problem. Our team has successfully induced LG dysfunction in model mice using scopolamine in the early stage [72]. On this basis, we have completed the research on the treatment of LG dysfunction by transplanting human LG adult stem cells based on the conditional reprograming technique. The research results are pending publication. Recently, Gleixner et al. [73] described the generation of a new immortalized human LG cell line by introducing the SV40 antigen, which provides more possibilities for further research in the future.  Table 1 summarizes all the published studies on the direct transplantation of stem cells and their products.

### 3.2. More Effective Drug Delivery Methods of Stem Cells and Stem Cell Products

Although the benefits of stem cell and extracellular vesicle regenerative therapies are widely known, direct transplantation entails risks associated with invasive procedures and issues regarding injection loss. Specifically, it is challenging to accurately control and guarantee the number of cells or products that can actually reach the effective target sites, thereby making it difficult to attain the anticipated effective treatment dosage.

Beyazyildiz et al. [74] found that eye drops might be an effective way to deliver MSCs to the eyes. After applying eye drops daily for a week, the labeled MSCs were detected in the conjunctival epithelium and meibomian glands of rats. Meanwhile, the tear secretion was also improved and the number of goblet cells increased. Similarly, Yu et al. [75] applied the extracellular vesicles secreted by human adipose MSCs locally as eye drops and found that their therapeutic effects were equally remarkable as those of MSCs eye drops. This indicates that the local application of MSCs or products through eye drops could be a safe and effective method for treating ADEE, and it has the potential to be used for further clinical research.

In addition, up to now, hyaluronic acid, gelatin, chitosan, and polypeptide-based hydrogels have been used to encapsulate extracellular vesicles from different cell sources [74]. For example, Methacrylated gelatin is a polymer with a loose porous structure that maintains the biological activity of the exosome and can control its slow release in vivo. Deng et al. [76] combined exosomes derived from human umbilical cord MSCs with methacrylated gelatin and applied them around venous grafts. It was demonstrated that this approach could accelerate reendothelialization after autologous vein transplantation and reduce restenosis in the rat model. This indicates that the development of more efficient drug delivery systems for LG stem cells and their products based on biomaterials could potentially be a promising therapeutic approach.

## 4. Organoid Therapy

Organoids are a type of model that is highly similar to the original tissues or organs in vivo and is established based on a three-dimensional (3D) in vitro cell culture system. For LG dysfunction diseases in which the LG is almost completely damaged, other therapies may not receive satisfactory results. It is necessary to utilize fully functional organoids constructed in vitro through 3D tissue reconstruction based on stem cells to complete the organ regeneration treatment [77].

### 4.1. Research on LG Organoid

Earliest, Hirayama et al. [78] cultured bioengineered LGs using embryonic germ cells by the organ embryogenesis method and transplanted them into LG-deficient mice. It was observed that they developed into acini and ducts and could produce tears in response to nerve stimulation. This study demonstrated the potential of organoid transplantation to functionally restore the LG. However, there are ethical issues regarding the acquisition and use of embryonic germ cells, making it difficult to achieve in clinical practice.

Subsequently, Bannier-Hélaouët et al. [79] constructed organoids based on LG stem cells, which could reflect the process of neurotransmitter-regulated tear secretion in vitro. After in situ transplantation in vivo, mature LG products were produced, thereby inducing crying. However, this LG organoid differentiates into tear-producing cells only by amplifying ductal stem cells and lacks acinar stem cells.

Conversely, Jeong et al. [80] established lacrimal organoids from human and mouse lacrimal tissues are more similar to acinar cells than to ductal cells in lacrimal tissues. The organoids established in their study recapitulate the structure and function of human LGs. Additionally, they developed LG organoids from Sjogren's syndrome patients (SS) and suggested that their developed SS organoids could be a useful tool as an in vitro model of mimetic SS disease. Finally, they preliminary demonstrated that the LG organoid possessed a self-repair effect of stem cells in an inflammation-induced DED animal model.

By comparison, Hayashi et al. [81] established 3D LG organoids with ductal and acinar stem cells from human induced pluripotent stem cells (hiPSCs). They cultured in Matrigel and a medium rich in epidermal growth factor, keratinocyte growth factor, fibroblast growth factor 10, bone morphogenetic protein 7, and Y-27632, which are the crucial factors required for the development of the LG. The organoids exhibit notable similarities to native LGs on the basis of their morphology, immunolabelling characteristics and gene expression patterns, and undergo functional maturation when transplanted adjacent to the eyes of recipient rats, developing lumina and producing tear-film proteins. This suggests that the transplantation of LG organoids can become an important treatment method for LG dysfunction diseases.

Similarly, Asal et al. [82] generated functional LG organoids in vitro using hiPSCs by adopting the multizonal ocular differentiation approach. However, the method used in their study is simpler and more concise, enabling the generation of functional cells in as little as 7 weeks. Moreover, they further showed derivation of lacrimal tissue specific cells and revealed metabolomic profiling of the functional LG organoids.

Recently, Nguyen et al. [83] further explored the signaling pathways affecting adult acinar cell expansion to optimize the organoid culture formulation, whereas R-spondin-enhanced pathway activation is known to have been used for expanding ductal cell populations as organoids from mouse and human tissues, with adult acinar cell expansion still presenting a challenge. They explored the ability of their antibody based WNT mimetic platform to stimulate LG tissue regeneration and founded murine acinar cell expansion as organoids by pathway activation through FZD1, 2,7. They demonstrated a role for WNT signaling in acinar cell proliferation.

Nevertheless, the 3D cell culture of the LG generally relies on Matrigel sourced from animals. The ambiguity regarding its components and the potential immunogenicity have hindered its application in human clinical transplantation [84]. In view of these limitations, there is a need to develop organoid culture methods that are independent of Matrigel.  Table 2 summarizes the researches on LG organoid.

### 4.2. New Advances of Matrigel-Free Organoid Culturing Methods

The cultivation methods which are based on acellular extracellular matrix, hydrogel, suspension culture, as well as microfluidic chip technology are prospective matrix-free ways to produce and sustain organoids [85].

Lin et al. [86] prepared LG scaffolds through decellularization and then used the cultured adult rabbit LG progenitor cells. In the 3D culture system, duct-like and acinus-like structures were formed. Moreover, the LG epithelial cells demonstrated good cell viability, cell differentiation and secretory function in the decellularized LG matrix. This study has proven the potential applicability of using tissue-derived acellular extracellular matrix for LG repair.

Recently, Wiebe-Ben Zakour et al. [87] developed a decellularized LG hydrogel that meets the requirements of bioink. Compared with the cells cultured on Matrigel, the proliferation of LG epithelial cells, MSCs and endothelial cells cultured with this hydrogel was enhanced, and the secretory capacity of LG epithelial cells was higher. To address the issue of its rapid biodegradation, the team used Genipin cross-linking to improve its stability, extend the culture period, and thus enhance its performance for in vitro culturing of LG organoids [88]. As a brand-new and more advantageous material, the decellularized LG hydrogel is expected to further promote the in vitro culturing of LG organoids and related research.

Microfluidic chips, with their precise control ability over fluids, can provide the physiologically required fluid shear forces, controllable growth factor concentrations, improve the supply of nutrients and oxygen, and promptly remove metabolites during the process of organoid culture [89]. This makes the organoids closer to the physiological characteristics of human organs. Lu et al. [90] established an in vitro 3D coculture model of the ocular surface, which consists of rabbit conjunctival epithelial and LG cell spheres, simulating the aqueous and mucin layers of the tear film and providing a more physiologically relevant treatment response. Although, some research teams are currently attempting to cultivate LG organoids using microfluidic chip technology, this field is still in a stage of continuous exploration.

### 4.3. New Advances in 3D Bioprinting Technology of Organoids

However, the currently constructed LG organoids can only achieve a basic similarity to the development and function of the human LG. Cultivating LG organoids that are more similar to the microstructure of the human LG is the key to success. Fortunately, magnetic 3D bioprinting technology and microfluidic 3D bioprinting technology have stepped in, offering novel concepts and highly promising solutions to address this challenge.

#### 4.3.1. Magnetic 3D Bioprinting Technology

Magnetic 3D bioprinting technology utilizes biocompatible magnetic nanoparticles (MNP) to tag cells. Subsequently, these labeled cells are printed into the 3D spatial tissue structure in accordance with the magnetic field, enabling them to freely assemble into a controllable size within a relatively short culturing period [91]. Rodboon et al. [92] have demonstrated that LG cells can be bioassembled into lacrispheres morphology through the magnetic 3D bioprinting platform. At 96 and 120 h, lacrispheres showed a significant improvement in cell viability over nonmagnetized LG cells in the same culture medium. Combined with the previous report that ovarian cancer cells cultured on the same platform were assembled into a papillary morphology, it indicates that the magnetic 3D bioprinting system not only allows magnetized cells to assemble in the presence of a magnetic field but also enables assembly through the spontaneous self-arrangement ability of each cell type [93].

Furthermore, Ferreira et al. [94] differentiated primary LG cells, which were then magnetically assembled into 3D spheroids, consistent 3D sphere-like cell clusters within 24 h with a stable ATP cellular production and viability. These spheroids exhibited a very consistent and uniform size over 3 days of culture. Histological examination and immunofluorescence staining revealed the structural and immunological similarities between LG spheroids and native LGs.

#### 4.3.2. Microfluidic 3D Bioprinting Technology

With the innovation of the microfluidic 3D bioprinting technology based on microfluidic technology, it is possible to precisely regulate fluids on a microscale and print droplets containing cells and related bioactive substances, according to the preset patterns and structures. This enables a more meticulous simulation of the arrangement of cells and the microenvironment within human tissue when constructing organoids [95].

Previously, the use of microfluidic platform in ophthalmology has been pioneered in studies mostly focusing on the cornea and retina [96]. While Koçak et al., [97] have generated a microfluidic platform for investigating the formation of the anterior segment of the eye derived from hiPSCs under various spatial-mechanoresponsive conditions. The organoids exhibited characteristics of multiple anterior segment ocular cell types including LG by immunofluorescence staining, but failed to show acinar and ductal structures.

**Figure 2:**
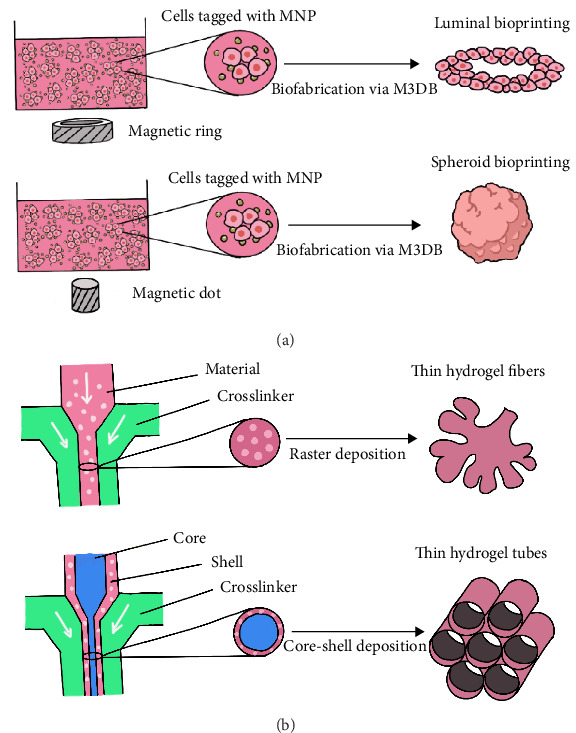
Magnetic 3D bioprinting technology and Microfluidic 3D Bioprinting Technology. (a) Magnetic 3D bioprinting (M3DB) employs magnetic nanoparticles (MNP) for luminal and spheroid bioprinting. (b) Microfluidic 3D bioprinting employs coaxial polymer and crosslinker streams to fabricate ultrathin hydrogel microfibers/tubes.

Unlike many other target tissues, for bioprinting glands uniquely rely on methods that can manufacture a thin epithelial layer. Yin et al. [98] exploited a microfluidics-based coaxial bioprinter with alginate as a model biomaterial for generating refined hydrogel structures with dimensions and morphologies inspired by salivary epithelia. They printed a broad range of accessible structure sizes, with reliable control over diameter for both fibers and hollow tubes, and wall thickness for tubes. Thin features like cell-laden microfibers or microtubes of physiologically relevant sizes can be successfully generated with ensured cell viability, retained stemness markers, and reliable reproducibility of feature sizes. It is well established that the LG and salivary glands exhibit a high degree of microstructural similarity. This bioprinting system may be a promising tool for advancing LG tissue engineering initiatives (Figure 2).

Although the above-mentioned 3D bioprinting technologies are still in the development stage at present and have their respective limitations, such as the biosafety of MNP, the impact on cell viability and function, and the long-term stability of the printed structures, which require further research and resolution. However, as emerging 3D printing technologies, they are expected to accurately simulate the microstructure of the human LG, which is helpful for culturing LG organoids that are highly similar to the human LG in terms of development, function, and microstructure, and thus have broad development prospects.

## 5. Conclusions

Our paper reviews the progress and prospects in the treatment of LG dysfunction diseases. It analyzes traditional treatment methods, including various treatment methods for alleviating the symptom of insufficient tear secretion (such as artificial tears, punctal plugs) and improving LG secretory function (such as biological tear substitutes, anti-inflammatory drugs, secretagogues, and salivary gland transplantation). It also points out their limitations and new progress, such as the combination of nanotechnology and anti-inflammatory drugs, as well as the research on allogeneic salivary gland transplantation.

Stem cell and organoid therapies are two emerging and cutting-edge research hotspots in the field of LG regeneration. At present, in research on stem cells or stem cell products, partial restoration of the secretory function and structure of the LG has been observed. The focus of further research in the future is to develop ophthalmic preparations of extracellular vesicles and LG stem cells or to search for more efficient drug delivery systems. Current studies on LG organoid have shown that after being transplanted, the organoids will go through processes, such as functional maturation, lumen development, and the production of tear-film proteins. The innovative development of new Matrigel-free methods, as well as the innovative development of magnetic 3D bioprinting technology and microfluidic 3D bioprinting technology, are expected to cultivate LG organoids that are highly similar to human LGs in both function and microstructure. This enables the full restoration of the structure and function of the injured LG, thereby bringing brand-new treatment hopes to numerous patients with LG dysfunctional diseases.

## Figures and Tables

**Table 1:** Direct transplantation of stem cells or stem cell products.

Type	Source	Author (year)	Model	Discovery	Advantages	Disadvantages
MSCs	Bone marrow	Lee et al. (2015) [52]	Mouse	Reduce the infiltration of CD4+ T cells and the levels of inflammatory cytokines	Discovered earliest	Not easy to obtain; high immunogenicity; quantity and quality of cells declining with age
		Aluri et al. (2017) [53]	Mouse	Relieve inflammation and increase the expression of aquaporin 5		
	Adipose tissue	Li et al. (2016) [55]	Rabbit	Improved autoimmune dacryoadenitis by regulating the Th1/Th17 response	Easiest and most abundant to obtain; low immunogenicity	Quantity and quality of cells declining with age
		Villatoro et al. (2015) [56]; Bittencourt et al. (2016) [57]	Dog	The long-term effectiveness of a single dose of a low number of allogeneic adipose-derived MSCs		
		Møller-Hansen et al. (2024) [58]	Human	Clinical trials of allogeneic adipose-derived MSCs transplantation are safe and feasible		
	Umbilical cord	Lu et al. (2020) [60]	Rabbit	Polarize macrophages into an anti-inflammatory phenotype through influencing the AKT pathway	Strong proliferative ability; low immunogenicity	Limited source of umbilical cord tissue; narrow time window for collection
	LG	Dietrich et al. (2019) [61]	Mouse	The number of acinar structures was increased and the immune reaction was also modulated	Tissue specificity	Limited source; poor differentiation potential

Extracellular vesicles derived from MSCs	Adipose tissue	Abuganam et al. (2019) [65]; Wang et al. (2023) [66]	Mouse	Extracellular vesicles have a similar effect on regeneration of the LG as MSCs do	Low immunogenicity; low tumorigenicity; convenient storage and transportation	Weaker self-renewal ability; poor durability
	Umbilical cord	Wang et al. (2023) [67]	Mouse			

LG stem cells	Progenitor cells	Gromova et al. (2017) [68]	Mouse	The effective use of adult LG epithelial progenitor cell transplantation	Safer; in line with ethics	Limited source; complicated amplification conditions
	Side population cells	Mishima et al. (2012) [69]	Mouse	Side population cells could also mediate the recovery of the hypofunctional LGs		

**Table 2:** Researches on LG organoid.

Source	Author (year)	Model	Discovery	Advantages	Disadvantages
Embryonic germ cells	Hirayama et al. (2013) [78]	Mouse	Develop into acini and ducts; produce tears in response to nerve stimulation	Differentiation potential	Ethical issues
LG stem cells	Bannier-Hélaouët et al. (2021) [79]	Mouse	Mature LG products are produced in vivo	First three-dimensional cell aggregate	Only amplify ductal stem cells and lacks acinar stem cells
Primary LG cells	Jeong et al. (2021) [80]	Mouse	Organoids possess a self-repair effect of stem cells in vivo	Further develop organoids from Sjogren's syndrome patients (SS)	More similar to acinar cells than to ductal cells
hiPSCs	Hayashi et al. (2022) [81]	Mouse	Undergo functional maturation; develop lumina; produce tear-film proteins	Develop into acini and ducts; similar to native LGs on morphology, immunolabelling characteristics and gene expression patterns	Rely on matrigel sourced from animals, lack similarity to the microstructure of the human LG
hiPSCs	Asal et al. (2023) [82]	—	—	Simpler and more concise culture methodology	
Primary LG cells	Nguyen et al. (2025) [83]	—	—	Demonstrate the role for WNT signaling in acinar cell proliferation	

## Data Availability

Data sharing is not applicable to this article as no datasets were generated or analyzed during the current study.

## Nomenclature

LG: Lacrimal gland
ADDE: Aqueous-deficient dry eye
DATS: Dynamic assessment of tear secretion
TMH: Tear meniscus height
MSCs: Mesenchymal stem cells
hiPSCs: Human-induced pluripotent stem cells
SS: Sjogren's syndrome patients
3D: Three-dimensional
M3DB: Magnetic 3D bioprinting
MNP: Magnetic nanoparticles.
